# Adhesive κ-Carrageenan Hydrogels by Polyphenol Intervention

**DOI:** 10.3390/biomimetics11040290

**Published:** 2026-04-21

**Authors:** Han-Yeol Yang, Jeongin Seo, Woongrak Choi, Eunu Kim, Sangho Yeo, Soeun Park, Haeshin Lee

**Affiliations:** 1 Department of Chemistry, Korea Advanced Institute of Science and Technology (KAIST), Daejeon 34141, Republic of Korea; 2 R&D Center, Polyphenol Factory Co., Ltd., Daejeon 34141, Republic of Korea; 3 Department of Pharmacy, Kyung Hee University, Seoul 02447, Republic of Korea

**Keywords:** κ-carrageenan, tannic acid, pyrogallol, degradation, adhesive hydrogel

## Abstract

Kappa-carrageenan (κ-CRG) forms thermo-reversible physical hydrogels via a coil–helix transition and helix bundling, but its sulfate-driven electrostatic repulsion limits mechanical robustness and control over aqueous disintegration. Here, we show that plant-derived polyphenols reprogram κ-CRG gel through sulfate-directed binding in a structure-dependent manner. Tannic acid (TA) selectively engages κ-CRG sulfate groups, yielding transparent gels and a >5-fold increase in storage modulus, whereas the same TA triggers turbidity and precipitation in sulfate-free agarose, supporting sulfate-mediated specificity. Using monomeric pyrogallol as a galloyl analogue, we demonstrate that monovalent interactions partially reinforce κ-CRG but lack cooperative stabilization. Intervention timing further separates mechanism. Pyrogallol produces pathway-dependent mechanics and gelation temperature, while TA is stage-insensitive, consistent with multivalent network annealing. In simulated gastric/intestinal fluids, pyrogallol/κ-CRG gels retain morphology longer, whereas TA/κ-CRG ones disintegrate rapidly yet exhibit strong adhesion to rough substrates and human skin. These findings provide a fully food-grade route to tune κ-CRG mechanics, thermal behavior, adhesion and programmed disintegration.

## 1. Introduction

Kappa-carrageenan (κ-CRG) is a sulfated galactan composed of repeating β-D-galactose-4-sulfate and 3,6-anhydro-D-galactose and extracted from red seaweeds that forms thermo-reversible physical hydrogels through a well-established mechanism [1]. At room temperature, κ-CRG exists as a self-supporting gel, whereas heating to around 90 °C disrupts the gel network and converts the polymer chains into a random-coil sol state. Upon cooling from this hot-sol state, individual chains undergo a random coil-to-helix transition, followed by aggregation of helices into junction zones that percolate into a three-dimensional network. This coil–helix transition and subsequently helix–helix association are highly sensitive to temperature, ionic environment, and polymer concentration, and they dictate the resulting gel stiffness, brittleness, and thermal hysteresis [2,3]. Owing to their biocompatibility and reversible gelation, κ-CRG hydrogels have been widely used as food thickeners and gelling agents [4], and have also been explored for drug delivery, wound dressings, and soft tissue engineering [5,6]. However, single-network κ-CRG gels are often mechanically fragile [7], show limited resistance to cyclic deformation, and offer only coarse control over degradation kinetics, which constrains their performance in demanding biomedical and cosmetic environments as well as in structured-food applications that require robust yet transient gels [8].

To overcome these limitations, considerable effort has been devoted to reinforcing κ-CRG-based hydrogels through chemical modification, double-network design [9], ion-mediated stabilization, and blending with synthetic or natural polymers such as poly(vinyl alcohol) (PVA) [10,11]. Recent studies and reviews continue to highlight the versatility of κ-carrageenan-based hydrogels in applications such as drug delivery, wound healing, and advanced biomaterial design, while also emphasizing the importance of controlling network stabilization and mechanical performance in these systems [12,13,14]. Although these approaches can enhance stiffness, toughness, and application-specific functionality, they may also require added salts, additional polymer networks, or complex formulations. This disconnect between mechanical reinforcement and formulation simplicity highlights the need for alternative strategies that preserve the naturally derived character of κ-CRG-based gels while enabling tunable mechanical and degradation properties. In this context, tannic acid (TA)—a plant-derived polyphenol composed of multiple galloyl units and widely present in foods and beverages—represents an attractive candidate crosslinker [15].

Beyond polysaccharide systems, TA has also been shown to act as a “molecular glue” for polyanionic biopolymers such as DNA. In a DNA hydrogel system termed “TNA gel” (TA + DNA), TA induces gelation by reversibly bridging the negatively charged phosphodiester backbone, without requiring complementary base-pairing or covalent modification of DNA [16]. In that design, the galloyl-rich architecture of TA mediates multivalent, non-covalent interactions with phosphate groups, yielding a hydrogel that is extensible upon pulling, adhesive to tissues, and intrinsically degradable because the ester linkages within TA gradually hydrolyze. This work demonstrated that a naturally derived polyphenol can directly engage an anionic backbone to construct mechanically robust yet degradable networks. Motivated by this precedent, we hypothesized that TA could similarly interact with the densely sulfated backbone of κ-CRG, forming sulfate-gallol physical crosslinks analogous to the phosphate–gallol interactions. On this basis, we set out to test whether such sulfate–gallol interactions could be harnessed to modulate both the mechanical reinforcement and degradation behavior of κ-CRG hydrogels.

Herein, we report a systematic investigation into how TA modulates the coil–helix transition, helix–helix association, and resulting network mechanics of κ-CRG hydrogels. We show that TA selectively interacts with the sulfate groups along the κ-CRG backbone, forming multivalent sulfate–galloyl physical crosslinks that tighten helix–helix junctions, increase junction-zone density, and substantially reinforce the three-dimensional gel network. To decouple the contribution of individual galloyl motifs from the multivalent architecture of TA, we further employed monofunctional pyrogallol as a comparison model. Unlike TA, pyrogallol alone is unable to generate extensive sulfate-bridging but instead partially inserts into nascent proto-helical regions, locally perturbing helix cohesion and altering thermal stability. This contrast highlights that the reinforcing capability of TA arises intrinsically from its multivalent galloyl organization rather than from galloyl chemistry alone. By integrating TA–κ-CRG interactions with pyrogallol-based comparisons, our study demonstrates that polyphenol–sulfate binding offers a previously underexplored and fully natural strategy to tune the microstructure, gelation kinetics, mechanical robustness, and thermal behavior of κ-CRG hydrogels without compromising their food-grade character.

## 2. Materials and Methods

### 2.1. Materials

κ-CRG (Genugel κappa-carrageenan type AKW; reported Mw range, 200–800 kDa) was purchased from CP Kelco (Atlanta, GA, USA). Tannic acid (≥96%; JIURUI Biology & Chemistry Co., Ltd., Hunan, China) was supplied by COSFINE Co., Ltd. (Gunpo, Republic of Korea). Pyrogallol (≥96%) was obtained from Sigma-Aldrich (St. Louis, MO, USA), and agarose (certified molecular biology grade) was obtained from Bio-Rad (Hercules, CA, USA). All reagents were used without further purification and deionized water (18.2 MΩ·cm, Milli-Q system, Millipore, Burlington, MA, USA) was used in all experiments.

### 2.2. Preparation of κ-CRG Gels

κ-CRG gels were prepared by dissolving κ-CRG powder in deionized water to a final concentration of 1% (*w*/*v*). This concentration was selected because it reliably formed self-supporting hydrogels with a suitable mechanical property, clearly exhibiting the effects of polyphenol addition on gel mechanics, thermal behavior, degradation, and adhesion. The solution was heated in a water bath at 90 °C or 70 °C until complete dissolution, then poured into designated molds and allowed to cool at room temperature to form gels. For κ-CRG gels containing additives, 2% (*w*/*v*) κ-CRG solution and aqueous solutions of TA or pyrogallol at desired concentrations were prepared separately and heated to the same temperature. The two solutions were then mixed at a 1:1 (*v*/*v*) ratio to obtain a final κ-CRG concentration of 1% (*w*/*v*). The resulting mixtures were cast into molds and cooled at room temperature to form gels.

### 2.3. Optical Characterization of Gel Transparency

For optical characterization, native κ-CRG, agarose only, TA (15%)/κ-CRG, TA (0.2%)/agarose, pyrogallol (6%)/κ-CRG, and pyrogallol (6%)/agarose gels were prepared directly in disposable cuvettes (1 cm path length). After gelation, their optical spectra were recorded using a UV–Vis spectrophotometer (HP 8453, Agilent Technologies, Santa Clara, CA, USA). The 500–600 nm region was used to compare relative transparency among the gel samples. For baseline correction, κ-CRG only and agarose only gels were used as blanks for the corresponding polyphenol-containing gel systems.

### 2.4. Rheological Characterization of Gel Networks

Gels prepared under different conditions were loaded onto the plate of a rheometer (MCR 302, Anton Paar, Graz, Austria) using a syringe. A parallel upper plate (25 mm diameter) was mounted, the gap was set to 1 mm, and the excess solution was trimmed. Rheological measurements were performed in frequency sweep mode, and the elastic behavior of the gels was analyzed based on the storage modulus (G′) measured at an angular frequency of 10 rad s^−1^. Prior to frequency sweep measurements, amplitude sweep tests were conducted to identify the linear viscoelastic region of each gel. A strain of 0.5% was then selected for all subsequent rheological measurements, as this value remained within the linear viscoelastic region for κ-CRG only, pyrogallol (6%)/κ-CRG, and TA (15%)/κ-CRG gels. To investigate synergy in mechanical properties when simultaneously adding TA (5%) + KCl (30 mM) compared with an individual addition either TA (5%) or KCl (30 mM), four κ-CRG (1%) gels (native κ-CRG, TA/κ-CRG, K^+^/κ-CRG, and K^+^/TA/κ-CRG) were prepared.

### 2.5. Fourier-Transform Infrared (FTIR) Analysis

For FTIR analysis, κ-CRG and κ-CRG:TA mixtures were prepared for spectroscopic comparison. FTIR spectra were collected using a Bruker Equinox-55 spectrometer (Bruker Optics, Ettlingen, Germany). Spectral changes were analyzed with particular attention to the D-galactose-4-sulfate region (840–850 cm^−1^).

### 2.6. Determination of Gel–Sol Transition Temperature

The gel–sol transition behavior was evaluated using temperature ramp measurements on the rheometer with a parallel-plate geometry. After loading the gel onto the lower plate, the gap was set and excess sample was trimmed. Measurements were conducted while heating from 25 °C to 90 °C at a constant rate (1 °C/5 s), with a fixed strain (γ) of 0.5% and angular frequency (ω) of 10 rad s^−1^. The normal force was maintained at 0 N throughout the measurement. Changes in G′ and G″ were recorded as a function of temperature, and the temperature at which G′ and G″ intersected was defined as the gel–sol transition temperature.

### 2.7. Disintegration Behavior in Simulated Gastrointestinal Conditions

κ-CRG (1% *w*/*v*) solutions containing either pyrogallol (6%, *w*/*w* relative to the κ-CRG solution) or TA (15% *w*/*w*) were dispensed into a 24-well plate (1 g per well, well diameter 15.5 mm). The solutions were allowed to cool at room temperature to form gels, yielding gel discs approximately 15.5 mm in diameter and ~5 mm in height. For the disintegration test, the gel discs were immersed in 5 mL of simulated aqueous media and incubated at 37 °C under agitation (100 rpm). Two media were used: simulated gastric fluid (SGF, pH 1.2) and simulated intestinal fluid (SIF, pH 6.8). The SGF solution was prepared by dissolving NaCl (2 g L^−1^) in deionized water followed by adjustment to pH 1.2 with 5 M HCl. The SIF solution consisted of 100 mM sodium phosphate buffer, with the pH adjusted to 6.8 using 5 M NaOH. The morphological changes and disintegration behavior of the gel discs were monitored over time. To quantitatively evaluate gel disintegration, the diameter of each gel disc was measured from top-view images taken at predetermined time points during incubation. The images were analyzed using ImageJ software (V1.54g, National Institutes of Health, Bethesda, MD, USA).

### 2.8. Adhesive Properties of Polyphenol/κ-CRG Gels

κ-CRG (1%), TA (15%)/κ-CRG (1%), and pyrogallol (6%)/κ-CRG (1%) gels were prepared using either cylindrical molds (35 mm diameter, 10 mm height) or rectangular molds (6545 mm^3^: 35 mm × 55 mm × 7 mm). The gel specimens were placed on sandpaper (P100) or on the forearm skin, after which the substrates were inverted to assess adhesion retention. If the gel remained attached after inversion, additional mechanical stimuli were applied by tapping the substrate or by shaking the arm vertically to evaluate forced detachment. For quantitative evaluation of adhesion strength, lap shear tests were performed using sandpaper (P100) as the adherend substrate. Sandpaper strips were cut into 70 mm × 20 mm pieces. The gel sample was placed between two sandpaper strips, which were overlapped over an area of 20 mm × 20 mm so that the gel was positioned at the bonding interface. The adhered assemblies were then tested using a universal testing machine (EZ-SX, Shimadzu, Kyoto, Japan) at a crosshead speed of 10 mm min^−1^. The maximum load at failure was recorded, and the lap shear adhesion strength was calculated by dividing the maximum load by the overlap area. The results were expressed in kPa.

All quantitative measurements were performed in triplicate, and the data are presented as mean ± standard deviation (SD).

## 3. Results and Discussion

### 3.1. Sulfate-Mediated Interaction of TA Governs Divergent Gelation Behaviors of κ-CRG and Agarose

**Figure 1:**
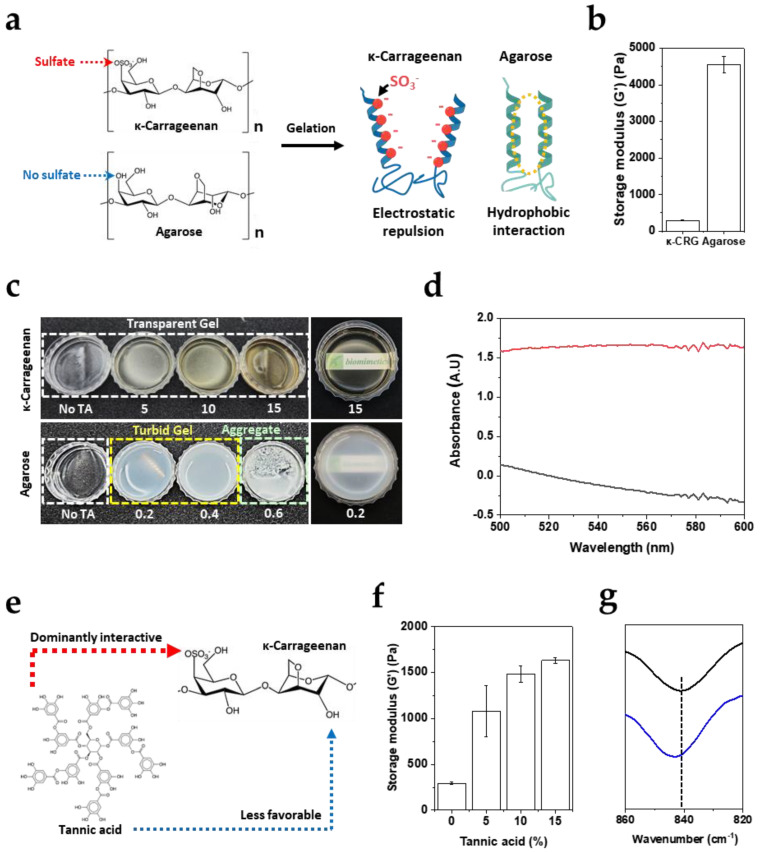
TA-mediated gelation behaviors of κ-CRG and agarose. (**a**) Schematic illustration comparing the molecular structures of κ-CRG and agarose. (**b**) Storage modulus (G′) of 1 wt% κ-CRG gels and 1 wt% agarose. (**c**) Optical appearance of κ-CRG and agarose gels prepared with increasing TA concentrations (wt%). (**d**) UV–Vis absorbance profiles of TA (15%)/κ-CRG (black) and TA (0.2%)/agarose (red) gels in the 500–600 nm region. (**e**) Proposed interaction scheme illustrating preferential binding of TA to sulfate groups in κ-CRG. (**f**) Storage modulus (G′) of κ-CRG gels as a function of TA concentration. (**g**) FTIR spectra of κ-CRG (black) and κ-CRG:TA ([κ-CRG]:[galloyl] = 1:14.7) (blue) in the D-galactose-4-sulfate region (840–850 cm^−1^), with the dashed line indicating 841.3 cm^−1^. Quantitative data in (**b**,**f**) are presented as mean ± SD (*n* = 3).

Figure 1 contrasts gelation behavior of κ-CRG and agarose because the two polysaccharides share a related galactan backbone structure yet differ critically in charge density. κ-CRG contains sulfate substituents that introduce strong anionic charges within networks, whereas agarose lacks sulfate groups. Thus, agarose gelation can rely effectively on helix–helix association supported by nonpolar (hydrophobic) interactions and dense hydrogen-bonding (Figure 1a). Although κ-CRG undergoes gelation through a similar helix-based association mechanism, electrostatic repulsion arising from its sulfate groups is expected to hinder close helix–helix packing and thereby reduce overall mechanical robustness relative to agarose. Consistent with the structural difference, the storage modulus (G′) of a 1% agarose gel is substantially higher (4560 ± 223 Pa) than that of a 1% κ-CRG gel (294 ± 10 Pa) (Figure 1b), indicating that agarose forms a rigid percolated network under the same concentration. These baseline data establish κ-CRG as a mechanically weaker but highly tunable platform whose modulus is constrained by charge-driven repulsion.

**Figure A1:**
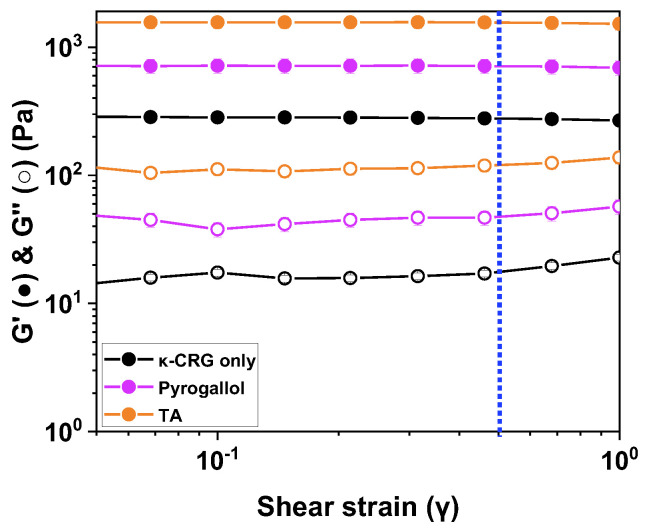
Amplitude sweep curves of κ-CRG only, pyrogallol (6%)/κ-CRG, and TA (15%)/κ-CRG gels to determine the linear viscoelastic region. Filled circles and open circles represent G′ and G″, respectively, for each formulation. The dashed blue line indicates the selected strain (0.5%) used for subsequent rheological measurements. Data are presented as mean ± SD (*n* = 3).

To study how TA influences the gelation of the two polysaccharides, TA was introduced over a broad concentration range. For agarose, the incorporation of only trace amounts of TA (0.2–0.4 wt%) was sufficient to produce turbid gels, and a slightly higher TA content (0.6 wt%) completely inhibited gel formation, yielding precipitated aggregates instead (Figure 1c, lower row). This behavior indicates that TA interacts strongly with the agarose galactan backbone, and that even a modest increase in TA concentration can disrupt the intrinsic helix–helix domain formation required for agarose gelation, thereby driving macroscopic phase separation and aggregation. In contrast, κ-CRG retained its characteristic transparent gel state even in the presence of 5 wt% TA—approximately ninefold higher than the TA level that abrogated agarose gelation (Figure 1c, upper row). Further increasing TA up to 15 wt% did not measurably compromise gelation or optical clarity. The optical distinction between the two systems was further supported by UV–Vis measurements, which confirmed optical transparency in TA/κ-CRG (A_550_ = −0.16) and pronounced turbidity in TA/agarose (A_550_ = 1.66) (Figure 1d). The slight negative value in absorption might be due to light scattering of solid gels. Because the principal structural distinction between agarose and κ-CRG is the presence of sulfate groups tethered to the backbone, the large difference in gelation/aggregation outcomes upon TA addition is most consistently explained by preferential TA binding with the sulfate functionalities in κ-CRG (Figure 1e). Such sulfate-directed binding would effectively sequester TA away from extensive direct binding to the neutral galactan backbone, thereby suppressing the rapid turbidity increase and precipitation observed in the agarose/TA system. We further quantified the viscoelastic reinforcement of the TA/κ-CRG hydrogels by measuring the storage modulus (G′) using an oscillatory rheometer (Figure 1f). All reported G′ values were obtained at a strain of 0.5%, which belongs to the linear viscoelastic region for all tested gel formulations (Figure A1). Notably, G′ increased in a TA concentration-dependent manner (5–15%), indicating progressive network strengthening with increasing TA content. In particular, at 15 wt% TA, the κ-CRG gel exhibited a G′ value more than fivefold higher (1632 ± 31 Pa) than that of the negative control (κ-CRG only) (294 ± 12 Pa). This mechanical enhancement is consistent with the interpretation that TA preferentially associates with κ-CRG sulfate groups, thereby partially neutralizing the intrinsic electrostatic repulsion between anionic helices. Reduced inter-helix repulsion would facilitate closer helix–helix packing and/or enlargement of junction zones, effectively increasing the crosslinking density of the physically crosslinked network and manifesting macroscopically as a higher storage modulus.

To provide evidence explaining the proposed sulfate-associated interaction between κ-CRG and the hydroxyl group of TA, FTIR spectroscopy was performed using κ-CRG and κ-CRG:TA mixture corresponding to [κ-CRG]:[galloyl] = 1:14.7. Under this condition, the peak position at 841.3 cm^−1^ was shifted to 843.2 cm^−1^ (Figure 1g). The range of wavenumber, 840–850 cm^−1^, belongs to the well-known region assigned to the sulfate attached to D-galactose [17]. These results support sulfate-associated intermolecular interactions between κ-CRG and the hydroxyl groups of TA.

### 3.2. Monovalent Galloyl Interactions Partially Reinforce κ-CRG Gels but Lack Cooperative Network Stabilization

**Figure 2:**
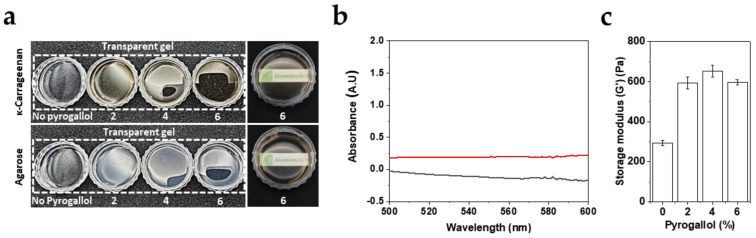
Effects of pyrogallol on the structure and mechanical properties of κ-CRG gels. (**a**) Optical appearance of κ-CRG and agarose gels prepared with increasing concentrations of pyrogallol. (**b**) UV–Vis absorbance profiles of pyrogallol (6%)/κ-CRG (black) and pyrogallol (6%)/agarose (red) gels in the 500–600 nm region. (**c**) Storage modulus (G′) of pyrogallol/κ-CRG gels as a function of pyrogallol concentration. Data are presented as mean ± SD (*n* = 3).

To distinguish whether the κ-CRG reinforcement observed with TA arises primarily from (i) additive, local charge screening at sulfate groups, or (ii) a multivalency-amplified, cooperative mechanism enabled by the clustered gallol units within TA, pyrogallol—a monomeric gallol lacking the multivalency of TA—was selected as a model small molecule and introduced at concentration of 2–6 wt%, chosen to provide gallol-equivalent inputs comparable to those used for TA. Qualitatively, both pyrogallol/κ-CRG and pyrogallol/agarose formed self-supporting, optically transparent gels across all tested pyrogallol concentrations (Figure 2a). Quantitatively, UV–Vis measurements showed A_550_ = −0.13 for pyrogallol/κ-CRG and A_550_ = 0.19 for pyrogallol/agarose (Figure 2b). Importantly, this result contrasts with the agarose/TA system shown in Figure 1c, where even trace amounts of TA induced turbidity and precipitation. The preservation of transparency and gel integrity in agarose upon pyrogallol addition suggests that monomeric gallol motifs, when presented without a multivalent scaffold, do not substantially disrupt helix formation or promote mesoscale complexation/phase separation in a neutral galactan matrix. In other words, the TA-driven turbidity and aggregation observed in agarose are consistent with multivalency-enabled bridging or condensation effects, rather than with a generic gallol–backbone interaction alone.

Consistent with selective strengthening of κ-CRG networks, oscillatory rheology revealed that pyrogallol increases the storage modulus (G’) of κ-CRG gels in a concentration-dependent manner, followed by an apparent saturation at higher pyrogallol loadings (Figure 2c). Relative to the pyrogallol-free control (~300 Pa), G’ increased markedly at 2–4 wt% pyrogallol to 600–700 Pa (593.15 ± 28.32 for 2 wt% and 653.21 ± 29.55 for 4 wt%) and then remained near this level at 6 wt%, indicating that a finite population of reinforcing interaction sites becomes progressively occupied and approaches a plateau (596.89 ± 12.72 Pa). These results support the conclusion that gallol motif alone can reinforce κ-CRG, plausibly associating with sulfate-rich domains and partially mitigating inter-helix electrostatic repulsion, thereby facilitating closer helix–helix association. However, when benchmarked against TA at comparable gallol equivalents (Figure 1f), pyrogallol yields substantially only a modest modulus enhancement, implying that charge screening by individual gallol units is not sufficient to recapitulate the full strengthening by TA. We attribute this gap to TA’s multivalency [18].

### 3.3. Polyphenol Intervention Stage Controls Pathway-Dependent vs. Pathway-Independent Gelation

**Figure 3:**
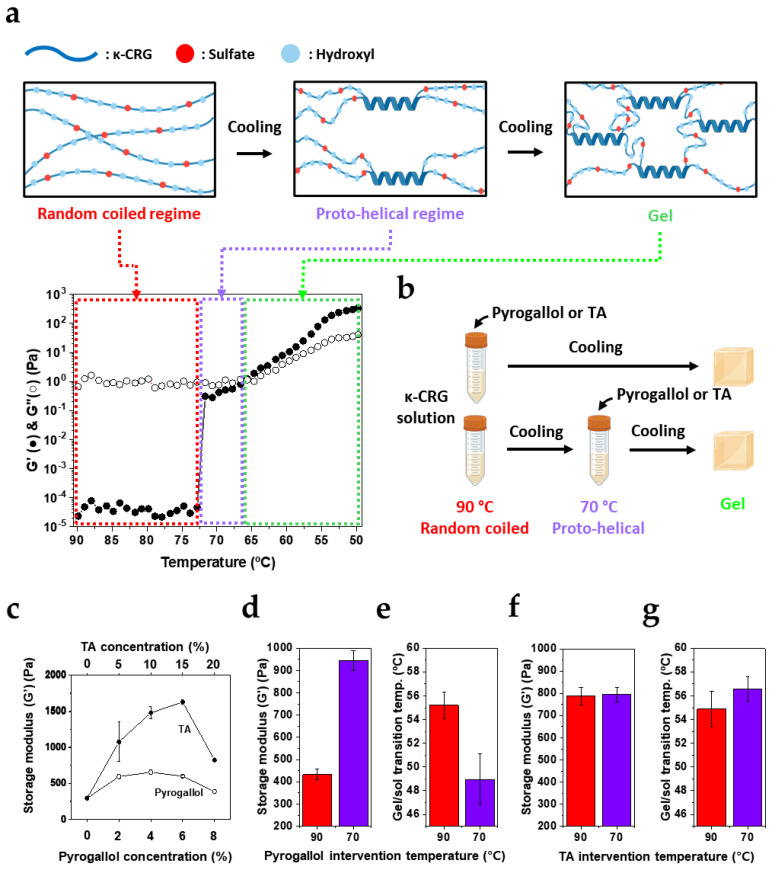
Stage-dependent effects of mono- and multivalent polyphenols on κ-CRG gelation. (**a**) Schematic illustration of κ-CRG gelation during cooling from a random-coil state to a proto-helical state and finally to a gel network, together with temperature-dependent rheological evolution defining the structural regimes. (**b**) Experimental design: polyphenols (Pyrogallol or TA) were introduced either in the random-coil regime (90 °C) or proto-helical regime (70 °C) prior to cooling and gel formation. (**c**) Storage modulus (G′) as a function of polyphenol concentration. (**d**) Storage modulus (G′) and (**e**) gel–sol transition temperature (°C) of pyrogallol. (**f**) Storage modulus (G′) and (**g**) gel–sol transition temperature (°C) of TA. Data in (**c**–**g**) are presented as mean ± SD (*n* = 3).

Gelation of κ-CRG proceeds through a stepwise process in which a high-temperature random-coil state undergoes a coil–helix transition upon cooling, followed by the development of inter-helical associations that form a three-dimensional network (Figure 3a, top). Because κ-CRG reinforcement observed with polyphenols in Figure 1 and Figure 2 was attributed to sulfate-associated interactions, we next examined whether the intervention stage—i.e., whether the polyphenol encounters κ-CRG before or during helix development—alters the final network architecture and properties. Temperature-ramp rheology revealed a narrow plateau for 1 wt% κ-CRG between 66.6 and 71.7 °C, which we define as a proto-helical regime, whereas ≥90 °C corresponds to a fully random-coil regime (Figure 3a). Accordingly, polyphenols (TA or pyrogallol) were introduced either at 90 °C or 70 °C while maintaining identical composition and processing otherwise (Figure 3b).

To select polyphenol concentrations for the temperature-dependent experiments (90 °C vs. 70 °C), we first monitored G’ as a function of polyphenol content. Across concentrations, both TA and pyrogallol exhibited a characteristic reinforcement-turnover profile. G’ increased at low polyphenol loadings, reached a maximum (~6 wt% for pyrogallol and ~15 wt% for TA), and then decreased at higher concentrations (Figure 3c). This trend is consistent with sulfate-mediated strengthening that eventually saturates, followed by structural perturbation of helix formation and/or junction-zone organization once excess polyphenol begins to frustrate helix–helix association. Based on these profiles, we selected 8 wt% for pyrogallol and 20 wt% for TA to clearly probe whether the final network depends on the stage of polyphenol intervention.

Strikingly, the intervention stage exerted a strong effect for pyrogallol but not for TA. When pyrogallol was introduced in the random-coil regime (90 °C), the resulting gel exhibited G′ = 432.5 ± 23.7 Pa after cooling to room temperature. In contrast, introduction of pyrogallol in the proto-helical regime (70 °C) produced substantially stiff gel G’ = 944.4 ± 44.9 Pa (Figure 3d). This result supports the view that κ-CRG is a weak gel, in which elasticity is governed by a subset of long-lived, load-bearing junction zones rather than network-average connectivity [19]. Importantly, the lower modulus upon pyrogallol addition at 90 °C (432.5 ± 23.7 Pa) can be rationalized by early-stage binding. Pyrogallol association with κ-CRG chains in the random-coil state likely perturbs subsequent coil → helix conversion and/or the organization of nascent helices into mechanically stable junction domains, thereby limiting the formation of load-bearing junction zones during cooling. By contrast, when pyrogallol is added after partial ordering has already emerged (70 °C), a fraction of proto-helical segments may be less accessible to disruptive binding [20], allowing junction-zone development to proceed more effectively and yielding higher G′ (944.4 ± 44.9 Pa).

Thermal signature of gelation corroborates this stage-dependent effect. Neat κ-CRG gelled at ~67 °C (Figure 3a), whereas pyrogallol addition depressed the gelation temperature to ~55 °C when introduced at 90 °C and further to ~49 °C when introduced at 70 °C (Figure 3e). The larger decrease upon 70 °C intervention suggests that, once proto-helical domains have formed, pyrogallol preferentially associates with the remaining exposed/less ordered chain regions, effectively modifying backbone interactions that are critical for propagating helix formation; consequently, helix development becomes less favorable, and gelation shifts to lower temperature. In contrast, TA produced little change in storage modulus (G’) and gelation temperature converged to a transition near 55 °C, comparable to the pyrogallol system mixed at 90 °C (Figure 3f,g). This stage-insensitive behavior is consistent with TA’s multivalency, which can promote chain-to-chain association through multipoint sulfate-associated interactions and thereby facilitate network organization even when introduced at different structural states [21]. Considering all results, Figure 3 demonstrates that monomeric pyrogallol imprints pathway dependence—with both stiffness and gelation temperature sensitive to the intervention stage—whereas multivalent TA behaves as a pathway-independent organizer, yielding similar thermal behavior while preserving robust network formation in κ-CRG gels.

### 3.4. Valency-Dependent Disintegration of Polyphenol/κ-CRG Gels in Aqueous Conditions

**Figure 4:**
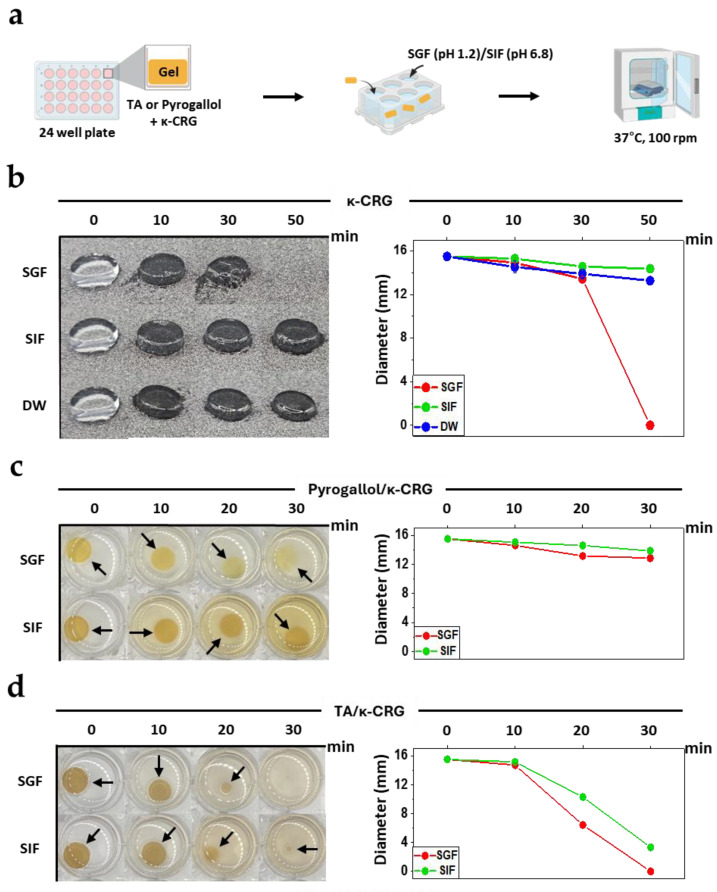
Disintegration behavior of κ-CRG gels in aqueous environments. (**a**) Schematic illustration of the experimental procedure. κ-CRG gels (15.5 mm diameter, 5 mm height) containing pyrogallol (6 wt%) or TA (15 wt%). Disintegration behaviors of κ-CRG only gels (**b**) in simulated gastric fluid (SGF), simulated intestinal fluid (SIF), and water (DW), pyrogallol/κ-CRG gels (**c**) in SGF and SIF, and TA/κ-CRG gels (**d**) in SGF and SIF. Arrows indicate the remaining gel discs at each time point. Quantitative results shown in (**b**–**d**) are presented as mean ± SD (*n* = 3).

Next, we assessed the macroscopic stability of the κ-CRG gel network in aqueous environments by monitoring the morphological evolution of gel discs over time. Gels were prepared by mixing pyrogallol (6 wt%) or TA (15 wt%) with hot 1 wt% κ-CRG followed by cooling, molding into discs (15.5 mm diameter, ~5 mm height), and incubating at 37 °C in simulated gastric fluid (SGF, pH 1.2) or simulated intestinal fluid (SIF, pH 6.8) with shaking (100 rpm) (Figure 4a). To quantitatively monitor the disintegration kinetics of the gel, the changes in gel diameter over time were additionally analyzed from top-view images using ImageJ (Figure 4b–d, right panels).

We first established the baseline behavior of native κ-CRG gels without polyphenols. The rate of disintegration followed SGF > DW > SIF (Figure 4b). In SGF (≈35 mM sodium), gel discs rapidly disintegrated within ~30–50 min, whereas in SIF the gels retained their structure over the same time scale and were even more stable than in DW. This qualitative trend was also supported by the diameter analysis. In SGF, the gel diameter decreased to 13.4 ± 0.2 mm at 30 min, and no measurable gel disc remained at 50 min due to complete disintegration, whereas the gels in SIF and DW retained diameters of 14.4 ± 0.2 mm and 13.3 ± 0.1 mm, respectively, at 50 min (Figure 4b, right panel). This pH/ionic-strength dependence is consistent with reduced junction stability at low pH, where partial protonation of sulfate groups diminishes anionic coordination sites important for helix aggregation [22], while the sodium-rich environment in SIF (100 mM sodium phosphate buffer) can partially stabilize helix-bundle junctions.

Upon polyphenol incorporation, a clear valency-dependent divergence in stability emerged, with pyrogallol-containing gels being consistently more stable than TA-containing ones in both SGF and SIF (Figure 4c,d). In both media, pyrogallol/κ-CRG discs underwent only gradual reductions in diameter over the observation window (0–30 min), preserving macroscopic integrity throughout. The gel diameter decreased from 15.5 to 12.9 ± 0.3 mm in SGF (Figure 4c, right panel, red) and from 15.5 to 13.9 ± 0.1 mm in SIF over 30 min (Figure 4c right panel, green). In contrast, TA/κ-CRG discs exhibited rapid shrinkage immediately upon exposure to aqueous media, with pronounced size reduction. In SGF, the diameter of TA/κ-CRG gels decreased from 15.5 to 14.7 ± 0.3 mm at 10 min, to 6.4 ± 0.2 mm at 20 min, and no measurable gel disc remained at 30 min (Figure 4d, right panel, red). Similarly, the diameter of TA/κ-CRG gels was decreased from 15.5 to 10.3 ± 0.1 mm at 20 min and 3.3 ± 0.3 mm at 30 min (Figure 4d, right panel, green). The observed faster breakdown of TA/κ-CRG gels can be rationalized by the distinct network architectures inferred from Figure 1, Figure 2 and Figure 3. Importantly, the increased initial G′ of TA/κ-CRG does not necessarily indicate greater resistance to aqueous disintegration. Pyrogallol primarily engages sulfate groups through localized, effectively monodentate interactions that partially reduce electrostatic repulsion yet preserve a substantial fraction of the native κ-CRG helix–helix junctions, allowing the network to retain global connectivity under hydrated conditions. In contrast, TA, owing to its multidentate architecture, promotes multipoint chain-to-chain bridging and dynamic bond exchange during gel formation [21], thereby markedly increasing gel stiffness while shifting network connectivity toward TA-mediated supramolecular junctions [23]. As a result, in aqueous environments, particularly in acidic SGF where native κ-CRG junctions are already destabilized, local dissociation or rearrangement of these TA-mediated junctions can directly disrupt percolation across the network and lead to rapid disintegration [24].

### 3.5. Multivalent TA-Mediated Surface Adhesion of κ-CRG Gels

The results presented in Figure 1, Figure 2, Figure 3 and Figure 4 demonstrate that polyphenol/κ-CRG interactions can reorganize the gel network from local molecular binding to the supramolecular architecture of the entire system. In particular, TA forms multipoint interactions between κ-CRG chains through its multivalent galloyl groups, generating supramolecular junctions that simultaneously modulate the mechanical properties of the gel and its disintegration behavior in aqueous environments. Such interactions are not necessarily limited to the internal network structure but may also operate at the gel–surface interface. Indeed, polyphenols are known to form strong non-covalent interactions with various polymeric and biological surfaces, particularly through polyphenol–protein and polyphenol–polysaccharide interactions [25,26].

**Figure 5:**
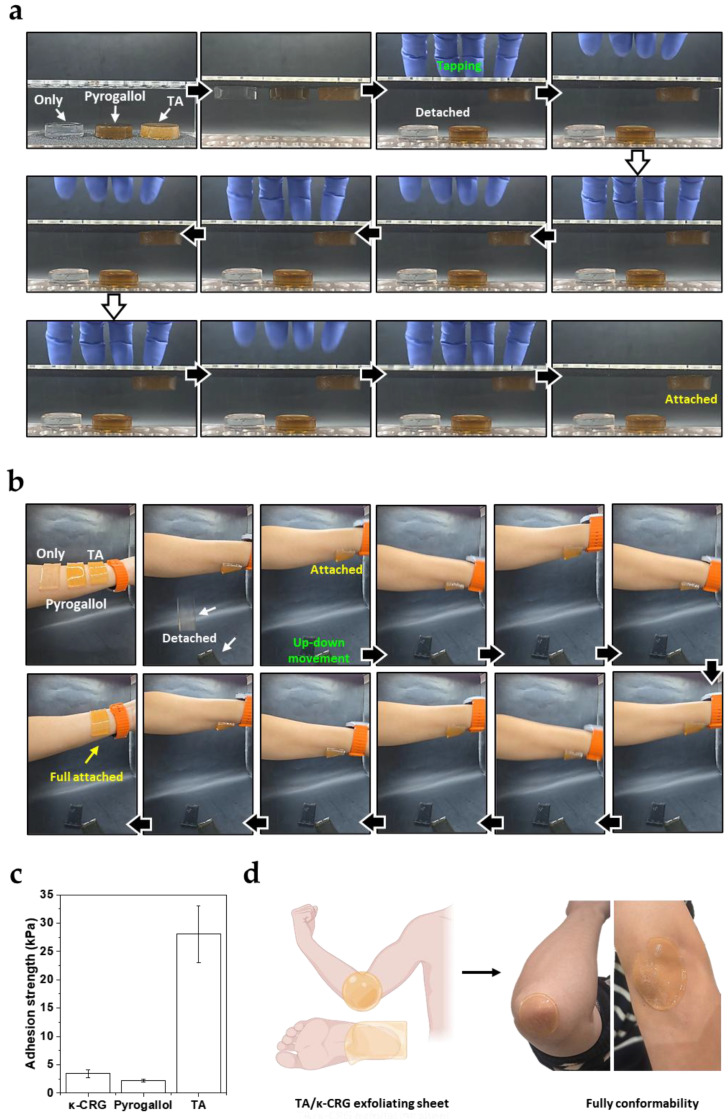
Surface adhesion and potential application of TA/κ-CRG gels. (**a**) Adhesion behavior of polyphenol/κ-CRG gels (35 mm diameter, 10 mm height) on a rough sandpaper (P100). (**b**) Adhesion behavior of gels (6545 mm^3^: 55 × 17 × 7 mm) on human skin. (**c**) Lap shear adhesion strength of native κ-CRG (left bar) and polyphenol/κ-CRG gels measured on sandpaper (P100). Data are presented as mean ± SD (*n* = 3). (**d**) Schematic illustration of a potential application of TA/κ-CRG gel as a conformable exfoliating sheet.

To test this hypothesis, the surface adhesion behavior of native κ-CRG gel, pyrogallol/κ-CRG gel, and TA/κ-CRG gel was compared. The gel discs were first placed on a rough sandpaper surface (P100), and their adhesion behavior was observed after the substrate was inverted so that the gels faced downward along the direction of gravity (Figure 5a). Both the native κ-CRG gel and the pyrogallol/κ-CRG gel detached immediately upon inversion. In contrast, the TA/κ-CRG gel remained firmly attached to the surface even when the substrate was completely inverted. The adhesion persisted even under repeated mechanical perturbations applied by tapping the substrate. These results indicate that the TA-containing gel forms stable interfacial interactions with rough solid surfaces. A similar trend was observed on human skin (Figure 5b). When gel discs of identical composition were attached to the skin between the wrist and forearm, the native κ-CRG gel and pyrogallol/κ-CRG gel detached immediately. In contrast, the TA/κ-CRG gel remained adhered to the skin surface and maintained its attachment even after repeated arm movements. This observation demonstrates that the TA/κ-CRG gel exhibits strong adhesion not only on rigid substrates but also on compliant biological surfaces such as skin.

**Figure A2:**
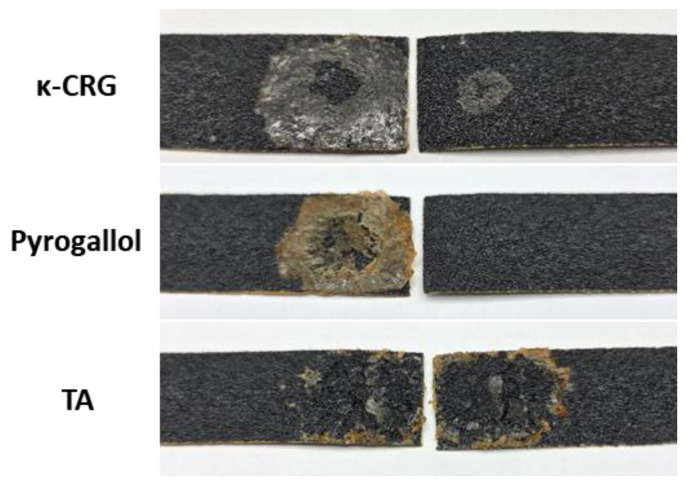
Failure mode after lap shear testing of κ-CRG, pyrogallol (6%)/κ-CRG, and TA (15%)/κ-CRG gels on sandpaper (P100).

To quantitatively evaluate this adhesion behavior, lap shear tests were performed using sandpaper (P100) as the adherend substrate (Figure 5c). The shear strengths of native κ-CRG, pyrogallol/κ-CRG, and TA/κ-CRG gels were 3.4 ± 0.7, 2.2 ± 0.3, and 28.1 ± 5.0 kPa, respectively. Consistent with the qualitative adhesion results, TA/κ-CRG exhibited markedly higher adhesion strength than either native κ-CRG (~3.4 kPa) or pyrogallol/κ-CRG (~2.2 kPa). Inspection of the sandpaper surfaces after lap shear tests further revealed distinct failure modes (Figure A2). Native κ-CRG and pyrogallol/κ-CRG primarily exhibited adhesive failure at the gel–sandpaper interface, whereas TA/κ-CRG showed cohesive failure within the gel layer due to the aforementioned strong adhesion.

The origin of this adhesion can be attributed to the multivalent nature of TA. Even after forming the gel network with κ-CRG chains, a fraction of galloyl groups may remain exposed at the network surface. These exposed galloyl groups can interact with opposing surfaces through multipoint non-covalent interactions, thereby promoting stable interfacial adhesion. Such multivalent interfacial interactions allow the gel to maintain stable adhesion even under mechanical perturbations [27]. The large increase in lap shear adhesion strength observed for TA/κ-CRG is consistent with this interpretation and further supports that TA-mediated multivalent interactions operate not only within the bulk gel network but also at the gel–substrate interface. This behavior is consistent with the dynamic supramolecular network characteristics of TA-mediated junctions discussed in Figure 3 and Figure 4.

The observed interfacial adhesion also suggests potential applications of TA/κ-CRG gels. When fabricated as thin sheets, the gels can form conformal coatings that adhere closely to skin surfaces. Through polyphenol-based interactions, the gels may effectively interact with biological macromolecules such as keratin in the stratum corneum. On this basis, the gels could potentially be utilized as exfoliating sheets that adhere to the skin surface (Figure 5d). In particular, the well-known protein affinity of TA [28] suggests that interactions with keratin may facilitate the removal of dead skin cells during sheet detachment. From a translational perspective, the TA content in skin-contacting κ-CRG gels should be selected by balancing interfacial adhesion and functional performance against possible irritation risk and cytocompatibility limitations, because tissue responses to TA can depend strongly on dose, formulation composition, and exposure conditions [29]. Although TA has been widely explored in skin-interfacing hydrogels owing to its polyphenol-mediated adhesion and additional antioxidant, antibacterial, and anti-inflammatory functions, these beneficial properties do not eliminate the need for careful dose optimization in topical formulations [15,28,30]. Prior dermatological literature has reported contact reactivity to topical tannic acid, with patch-test positivity observed at concentrations as low as 0.25% (aq.) in susceptible cases [30]. At the same time, the biological response to TA is likely formulation-dependent, and TA embedded within a polymeric network may behave differently from free TA applied directly to skin. Therefore, further studies will be required to define an appropriate TA dose window that preserves adhesion while minimizing barrier perturbation and irritation, ideally through standardized cytocompatibility assays, reconstructed epidermis or barrier-function testing, and human patch testing before biomedical or cosmetic translation.

**Figure A3:**
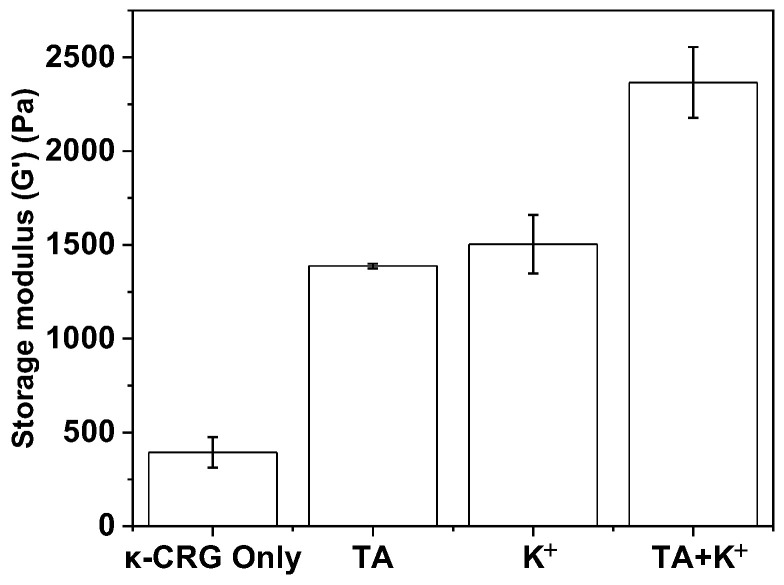
Storage modulus (G′) of κ-CRG only, TA (5%)/κ-CRG, K^+^ (30 mM)/κ-CRG, and TA (5%)/K^+^ (30 mM)/κ-CRG gels. Data are presented as mean ± SD (*n* = 3).

In perspective of the context of existing κ-CRG reinforcement strategies, the present results show that polyphenol-mediated modulation of κ-CRG gels differs in character from conventional strategies used to control carrageenan gel networks. Existing approaches often rely on ion-mediated stabilization, such as K^+^- or Ca^2+^-assisted helix aggregation and junction-zone stabilization [31,32], or on polymer-based reinforcement through blending with additional polymers such as PVA, double-network design, or formation of an additional polymer-supported matrix [9,10,11]. By contrast, the present gelation strategies utilize small molecule polyphenols (pyrogallol = 126.1 Da and TA = 1701.2 Da) as new additives to form sulfate–polyphenol–sulfate bridges keeping the inherent helical domains intact [16]. Thus, the binding mode differences between K^+^ and TA are allowed synergistic mechanical benefits in case of simultaneous addition of K^+^ and TA. Upon the addition of potassium ion, the storage modulus of the κ-CRG gel was increased from 393.8 ± 81.6 to 1503.2 ± 156.1 Pa, which is similar to that of the TA/κ-CRG gel (1387.2 ± 12.7 Pa). Notably, additions of both TA (5%) + K^+^ (30 mM) exhibited further increase (2366.1 ± 189.4 Pa) (Figure A3). This result indicates that TA-mediated modulation should be viewed not simply as another reinforcement additive, but as a distinct route for controlling the structure–property relationships of κ-CRG hydrogels.

## 4. Conclusions

In this study, we investigated how polyphenols with different valencies influence the structure, mechanics, and macroscopic behavior of κ-CRG hydrogels. By comparing TA and pyrogallol, we demonstrated that polyphenol valency plays a key role in regulating κ-CRG gel networks. Both polyphenols interact with κ-CRG through non-covalent interactions involving sulfate groups, which partially reduces electrostatic repulsion between helices and reinforces the gel structure. However, the extent and consequences of these interactions differ markedly depending on the polyphenol architecture. Pyrogallol produced moderate reinforcement and exhibited stage-dependent effects during gelation, indicating that its interaction with κ-CRG is sensitive to the conformational state of the polymer chains. In contrast, TA generated substantially stronger network reinforcement and showed stage-independent gelation behavior owing to its ability to form dynamic multivalent supramolecular junctions between polymer chains. These differences also resulted in distinct macroscopic degradation responses in aqueous environments. Pyrogallol/κ-CRG gels maintained structural integrity longer than TA/κ-CRG gels. Interestingly, TA/κ-CRG gels exhibited strong adhesion on rough, and biological surfaces, highlighting the interfacial functionality enabled by multivalent polyphenol chemistry. Overall, our findings reveal that polyphenol valency is a critical molecular parameter governing the mechanics, stability, and interfacial properties of κ-CRG hydrogels. This work provides new insights into polyphenol–polysaccharide interactions and offers a practical strategy for designing food-grade functional hydrogels with tunable mechanical and adhesive properties.

## Data Availability

The data presented in this study are available from the corresponding author upon reasonable request.
